# In Situ Bacterioplankton Growth Partitioning by High‐Resolution Metatranscriptomics

**DOI:** 10.1111/1758-2229.70385

**Published:** 2026-07-09

**Authors:** J. Cesar Ignacio‐Espinoza, Yuxuan Zou, Andrew Long, Shengwei Hou, David M. Needham, Jed A. Fuhrman

**Affiliations:** ^1^ Marine and Environmental Biology, Department of Biological Sciences University of Southern California Los Angeles California USA; ^2^ State Key Joint Laboratory of Environmental Simulation and Pollution Control, School of Environment Beijing Normal University Beijing China

## Abstract

Microbial communities are fundamental to marine trophic webs, elemental cycling and geochemical transformations. Yet we lack high phylogenetic resolution measurements or good indicators of how fast different taxa are actively growing in situ. Here, we use high temporal resolution transcriptomics to phylogenetically disentangle an index of growth before and after a short spring phytoplankton bloom, by quantifying the phylogenetic distribution of the expression of *ftsZ*, a gene encoding for a protein involved in cell division. We interpret the relative abundances of *ftsZ* transcripts as a general indicator of how growth was distributed amongst taxa. This expression was also compared to RNase P to estimate how each organism's transcriptional resources were allocated to replication versus other functions. During the time‐series, we observed two distinct profiles: prior to and several days after the bloom, *ftsZ* expression was dominated by *Synechococcales*, *Pelagibacterales* and picoeukaryotes, and by *Rhodobacterales*, SAR92 and SAR86 as the bloom declined. Whilst similar successional patterns have been observed previously, our dataset extends these observations by resolving transcriptional and replication‐associated activity at high temporal resolution, enabling the detection of disproportionate contributions during bloom development and turnover. Our approach is scalable and will inform conceptual and mechanistic models of planktonic food webs.

## Graphical abstract

Bacterial growth and primary production are key ecological indicators, yet often measured in bulk. Using metatranscriptomics, the authors identify *FtsZ* expression to track dividing cells (A, B). Phylogenetic placement of transcripts (C–E) enables linking growth activity to specific taxa (E, F), potentially bridging bulk measurements with fine‐scale ecological resolution across microbial communities.

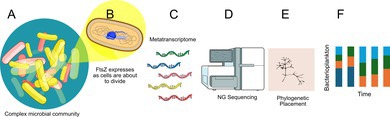

## 1 Introduction

Understanding microbial growth and activity in natural environments is essential for gaining a mechanistic understanding of elemental cycles and the flow of energy and matter in the food webs of our changing oceans (Kirchman 2016). Although we often wish to know how different members of the community contribute to primary and secondary (heterotrophic) production, most traditional methods have relied on bulk approaches to get total primary or secondary production, such as measuring the rate of incorporation of isotopically labelled compounds (Fuhrman and Azam 1982; Kirchman et al. 1985). These approaches treat microbial assemblages as a whole and do not discern contributions of individual phylogenetic groups to community‐wide rates. Moreover, most of the expectations about differential taxa‐specific growth in nature stem from our knowledge of cultured representatives, raising questions about their accuracy in reflecting naturally occurring rates, especially considering that we currently have cultures of only a tiny fraction of natural microbial diversity. Moreover, cultivation tends to select for faster‐growing (copiotrophic) microbes (Weissman et al. 2021).

There is a longstanding discussion about how the activity spectrum from microbial dormancy to rapid growth maps onto the spectrum of abundant to rare prokaryotes in natural marine systems (Pedrós‐Alió 2006; Jones and Lennon 2010; Campbell et al. 2011). To address these challenges, some approaches with the goal of assigning activity rates to phylogenetically narrow groups have been developed. These include autoradiography with fluorescence in situ hybridization and ratios of the 16S rRNA:rDNA (Campbell et al. 2011; Hunt et al. 2013), the latter based on the observation that many microorganisms have proportionally more RNA when growing rapidly. However, whilst cellular rRNA (Calabrese et al. 2024) often scales according to growth rates, this broad generality cannot always be applied (De Vrieze et al. 2016; Blazewicz et al. 2013). Furthermore, in practise, rRNA and rDNA are typically each measured compositionally (i.e., each organism as a fraction of the total RNA or DNA) and the unambiguous interpretation of the ratios of such fractions is difficult.

Cellular division is a key indicator of microbial activity, and the expression of certain genes, such as *dnaA* and *ftsZ*, closely follows the cell cycle (Holtzendorff et al. 2001, 2002). Previous studies have used this premise to quantify and measure *ftsZ* expression in both cultured (Holtzendorff et al. 2001) and natural environments (Holtzendorff et al. 2002; Yao et al. 2011) as an indicator of microbial activity. These studies developed quantitative PCR (qPCR) experiments targeting the *ftsZ* gene (Holtzendorff et al. 2002; Yao et al. 2011), which encodes for a protein that forms part of the cell membrane's inner ring during cell division. As a result, *ftsZ* expression is tightly linked to the preparation for cell division (Margolin 2005; Pando and van Oudenaarden 2010), making it an excellent marker for microbial growth monitoring. Whilst multiple genes (including *dnaA*) are involved in the replication and division cycle, here we specifically focus on *ftsZ* due to its prior use and strong association with cell division across diverse taxa. However, qPCR assays in those studies were limited to a few targeted taxa, as the method requires the design of specific primers for each organism of interest (Holtzendorff et al. 2002; Yao et al. 2011). This makes broad taxonomic coverage challenging, especially in complex environmental samples where many microbial groups may be unknown, uncultured, or poorly annotated. In the present work, we expand on this approach by employing whole‐community RNA sequencing to detect *ftsZ* mRNA transcripts. These samples were previously analysed by Needham et al. (2018), who characterised microbial succession using 16S and 18S rRNA genes and rRNA:rRNA‐gene ratios as proxies for relative abundance and potential activity. In contrast, our current work focuses specifically on replication‐associated transcription through phylogenetically resolved *ftsZ* expression. Thus, rather than re‐characterising overall community composition, our approach aims to determine how growth‐related transcriptional investment was distributed amongst taxa during bloom‐associated succession.

## 2 Experimental Procedures

### 2.1 Sampling

We collected and filtered seawater using an Environmental Sampling Processor (Scholin et al. 2009) (ESP) tethered to the seabed off the coast of Southern California, near the San Pedro Ocean Time Series (Cram et al. 2015) study site (33°28.990′N, 118°30.470′W). Our instrument was deployed from 18 March to 1 May of 2014; however, due to a temporary instrument failure, sampling did not occur for approximately 2 weeks (23 March–8 April). Before the interruption, sampling occurred daily at 10:00 a.m. and after the interruption, sampling was conducted twice daily at 10:00 a.m. and 10:00 p.m., during which 1 L of seawater was collected. Other measurements were also taken; see Needham et al. (2018) for detailed information.

Since the instrument was tethered to the bottom of the ocean by a long cable, it was free to float within the water column, causing the sampling depth to vary throughout the experiment, ranging from 5 to 15 m. However, the sampling predominantly occurred within the mixed layer where physical mixing typically homogenises microbial communities and their transcriptomic profiles. Water samples were first pre‐screened through a 300 μm copper screen and then sequentially filtered through a 1 μm AE glass filter (Pall Gellman) and a 0.22 μm Durapore polyvinylidene filter (Millipore). All filters were stored in RNAlater at ambient temperature until the instrument retrieval, after which they were stored at −80°C until molecular processing. Nucleic acids were extracted from both filters and analysed elsewhere (Needham et al. 2018); for our current work we only generated transcriptomes from the second filter (1–0.2 μm), which is expected to predominantly capture free‐living prokaryotes.

### 2.2 Molecular Methods

For detailed methods, see Needham et al. (2018). Briefly, RNA was extracted from both filters using the RNeasy kit (Qiagen) according to the manufacturer's instructions, including the DNase digestion step. To avoid potential biases, RNA extractions were not depleted for ribosomal RNA nor enriched for messenger RNA. RNA was reverse transcribed to cDNA using SuperScript III (Invitrogen) with random hexamers, using 10 ng of total RNA. Single‐strand cDNA was then converted to double‐strand cDNA using the Second Strand cDNA synthesis kit (Invitrogen). The cDNA was then purified using magnetic beads and used as input for library preparation. Libraries were prepared using the Ovation Ultralow V2 DNA‐Seq library preparation kit (NuGEN, Tecan Genomics) under the manufacturer's instructions using 10 ng of starting DNA and amplified for 13 PCR cycles. Extraction, retrotranscription, or library preparation failed for a few samples, denoted as empty bars or cells in the corresponding figures and Supporting Information tables.

### 2.3 Sequencing and Sequence Analysis

Libraries were sequenced on an Illumina HiSeq 4000 system (2 × 150 bp) at the USC genomics core facilities. Sequences were demultiplexed, and quality was assessed using FastQC v0.11.2. Adapters, primers and low‐quality bases were removed using cutadapt v2.8 with the following ‘cutadapt –cut 15 ‐q 18’. Two phylogenetic trees were constructed using *ftsZ* full‐length sequences recovered from NCBI and RNase P sequences recovered from RFAM (Kalvari et al. 2021). Sequences were aligned using MAFFT (Katoh and Standley 2013) v7.3x, with default settings (‘mafft–auto’) and the trees were built using RaxML v8 with default settings (−m GTRGAMMA). Both, the tree and alignment were then used to create a pplacer ‘package’ (guppy package‐o—t‐s‐a). Then pplacer (Matsen et al. 2010) v1.0 was used for phylogenetic placement using the entirety of the metagenomic libraries (‘pplacer‐c’), pplacer calculates the likelihood of placement of a read on every edge of the tree, which can be either a leaf or an internal node (indicating for example a class or an order). Taxonomy of all nodes was extracted from annotations in both databases used above.

### 2.4 Data Aggregation, Analysis, and Representation

Read counts were aggregated at different taxonomic levels (see Table S1) to include assignments to internal tree nodes. These counts were scaled to relative abundances for data visualisation purposes only; raw counts were used for the time series analysis and expressed as log_2_ fold changes within samples. We applied linear mixed‐effects models (LMM) using the statsmodels package in Python. To assess the breadth of *ftsZ* fold changes amongst microbial taxa, we modelled the log‐ratio as a function of each microbe, treated as a fixed effect, with collection time included as a random intercept. To evaluate the specific effect of the diel cycle (day vs. night), separate LMMs were constructed for each taxon. In these models, the day/night status was a fixed effect and the specific sampling timepoint was included as a random intercept. *p*‐values for the day/night effect were adjusted for multiple comparisons using the Benjamini–Hochberg False Discovery Rate (FDR) method.

## 3 Results

### 3.1 Community Transcriptomics Recovers an Unbiased Landscape of Microbial Activity

**FIGURE 1:**
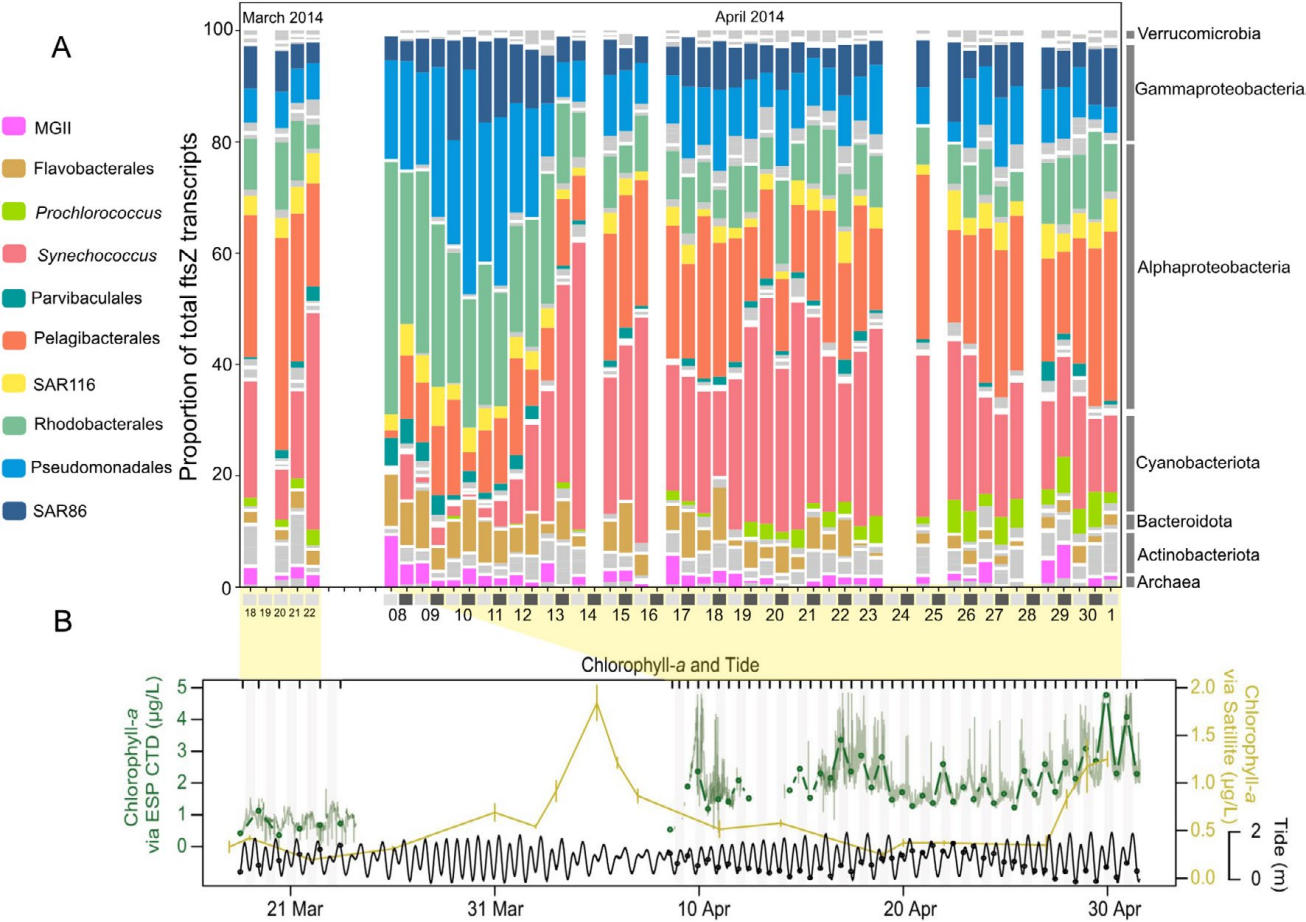
Temporal dynamics of *ftsZ* mRNA fragment abundance. (A) The horizontal axis represents collection dates from 18 March 2014 to 1 May 2014. Each bar illustrates the proportion of total *ftsZ* transcripts contributed by various microbial groups over time. Classes are marked with dark grey bars on the right, and members within a class are stacked on top of each other, separated by white lines. Most colours represent orders, except where higher resolution and data allow for further granularity (e.g., *Synechococcus* and *Prochlorococcus*). The clear and dark boxes at the bottom indicate collection times, either 10:00 or 22:00 h, respectively. For a more detailed breakdown of phylogenetic composition, see Table S1. Note the 2‐week data gap between March and April due to instrument failure. Although the peak of the spring phytoplankton bloom (April 5–6) was missed (B), shifts in microbial community structure, as indicated by changes in ftsZ abundance, are evident shortly after the instrument resumed function. Further details on the short‐lived bloom are provided in reference (Needham et al. 2018). Briefly (B), chlorophyll‐a concentrations from the ESP CTD (left) and satellite‐derived chlorophyll concentrations averaged over a 3 × 3 km area (right). Note that Panels A and B are shown on different scales to account for the gap caused by instrument failure.

We obtained ~1 million reads per sample after quality control and computational removal of rRNA for a total of 44.5 million reads from 45 metatranscriptomic samples of the prokaryotic size fraction (1.0–0.22 μm). A total of 9800 *ftsZ* fragments were identified by sequence similarity and phylogenetically placed using a high‐resolution, highly curated phylogenetic tree. Our data revealed that *ftsZ* expression was predominantly driven by members of 10 Classes of prokaryotes (Figure 1); with a small number of orders within a class and a few families within an order (Figure S1 and Table S1) accounting for the majority of transcriptomic *ftsZ* reads. We identified two distinct community profiles that corresponded to (i) before the onset and after this small bloom, likely reflecting a long‐term quasi‐equilibrium state and (ii) during the late bloom, where heterotrophic bacteria are consuming fresh organic matter derived from the declining bloom.

The *ftsZ* expression profiles during the pre‐bloom period (March) and towards the end of the study (after about 13 April) were dominated by *Synechococcales* (*Synechococcus* and *Prochlorococcus*) and *Pelagibacterales* (SAR11). In the days immediately following the bloom (8–13 April), the *ftsZ* expression was dominated by *Rhodobacterales*, SAR92 (*Pseudomonadales*) and SAR86. We interpret this as indicating these and other microbial groups responded to the bloom with increased growth because they are readily adapted to degrade phytoplankton‐derived organic matter, as reported previously (Teeling et al. 2016).

### 3.2 Chloroplast‐Derived *ftsZ* Sequence Fragments

Interestingly, a significant number of reads phylogenetically assigned to Eukaryotes were also recovered (up to 40%, Figure S1). These reads originated from chloroplasts, which encode, express and direct their own divisions, including the use of *ftsZ* (Margolin 2005). Chloroplast division parallels that of their containing cells, in some cases including checkpoints that ensure chloroplast division as the cell prepares to divide (Sumiya et al. 2016). Our current work focuses on the fraction of biomass between 1.0 and 0.22 μm, targeting primarily bacteria. The picoplanktonic eukaryotes in that fraction represent just a small portion of the total phytoplankton, so we lack the ability for direct comparisons with the rest of the eukaryotic phytoplankton. As organelles, the chloroplasts may not have expression rates and patterns directly comparable to the prokaryotic cyanobacteria, thus they were considered separately. Despite this caveat and others, such as variable numbers and lack of full synchronicity to the cell cycle, our findings suggest that chloroplast *ftsZ* expression, in whole‐community metatranscriptomics, may be useful to taxonomically partition growth and primary productivity amongst natural phytoplankton communities, using the chloroplast *ftsZ* as an indicator. This signal, whilst representing relative transcriptional contribution, opens a path for taxonomically informed partitioning of eukaryotic division processes.

### 3.3 Replication Versus Baseline Transcriptional Activity

RNase P is a crucial ribonucleoprotein complex that plays a significant role in the maturation of precursor tRNA molecules by cleaving their 5′ ends. It is widely recognised for its critical function across various domains of life, including bacteria, archaea and eukaryotes, making it both indispensable and ubiquitous (Kazantsev and Pace 2006; Walker and Engelke 2006). Due to its importance, necessary levels of this enzyme are maintained for proper cellular function, and it is known to be constitutively expressed in at least some taxa (Wicke et al. 2023) and in most bacteria forms part of the same operon encoding for ribosomal proteins (Ogasawara and Yoshikawa 1992; Feltens et al. 2003). We posit that the relationship between the expression of the *ftsZ* gene and that of the RNase P gene could provide a robust estimation of a cell's allocation towards cell division versus its baseline transcriptional activity. The expression of these genes can reflect different cellular priorities; *ftsZ* expression is indicative of active cell division (Weart and Levin 2003), whilst RNase P expression may represent baseline transcriptional activity necessary for cellular maintenance and function, given its conservation, essentiality and ubiquity (Kazantsev and Pace 2006; Walker and Engelke 2006; Wicke et al. 2023; Ogasawara and Yoshikawa 1992; Feltens et al. 2003). To this end, we calculated the fold difference between *ftsZ* and RNase P to indicate the proportion of a microbial clade's transcriptional resources allocated to replication. This calculation is analogous, although admittedly coarser, to that performed by modern RNA‐seq methods that use ‘housekeeping genes’ as source for scaling factors (Love et al. 2014). Whilst the set of genes involved in replication includes many additional markers beyond *ftsZ*, our decision to focus on this single, well‐characterised gene represents a practical and informative starting point. *ftsZ* offers a phylogenetically broad signal directly tied to cell division, allowing us to infer how organisms prioritise energy and resources towards replication versus basal maintenance. Unlike inferred growth rates derived from amplicon or metagenomic (even normalised to cell abundance (Deulofeu‐Capo et al. 2024) or internal standards (Fecskeová et al. 2021)) which typically reflect either gross or net growth, our work provides a complementary perspective. By quantifying transcript allocation to *ftsZ* relative to a housekeeping gene like RNase P, we begin to bridge the gap between first‐principles cellular physiology and emergent community‐level growth dynamics (Deulofeu‐Capo et al. 2024; Fecskeová et al. 2021; Calabrese et al. 2024).

**FIGURE 2:**
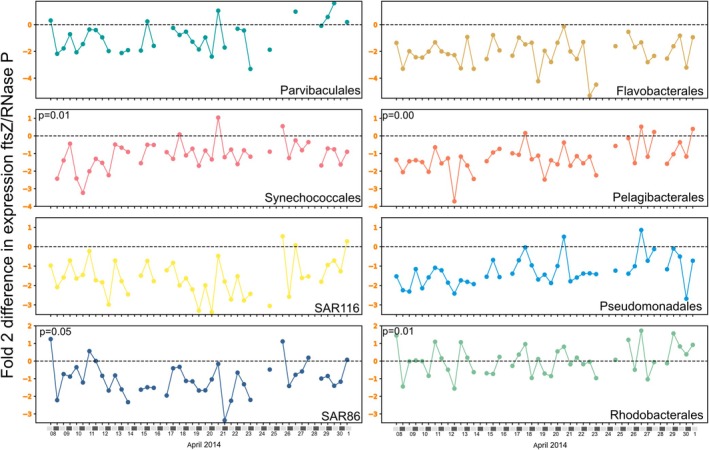
Fold differences in expression of *ftsZ* relative to RNase P over a time series from 8 April to 30 April 2014, across different bacterial orders. The y‐axis shows the fold difference in expression of the *ftsZ* gene normalised against RNase P, with values plotted on a log_2_ scale. A value of 0 indicates no difference in expression, whilst positive and negative values represent higher or lower expression of *ftsZ* relative to RNase P, respectively. Linear mixed models indicate significant differences amongst all eight groups across all time points, with significant day–night fold changes detected for only four of the groups (*p*‐values shown in the upper left corner). Missing data points correspond to either failed extraction, amplification or library preparation.

We observed day‐to‐day and day‐night variations in these *ftsZ*/RNaseP ratios plotted as fold changes (Figure 2). We used log_2_‐transformed fold changes, a common metric in transcriptomics dating back to microarray studies, which has also proven useful for compositional data. The observed temporal variation in these log_2_ ratios further supports the utility of *ftsZ* as a marker: since a less regulated gene would not behave in this manner. Analysing and aggregating these data provides informative insights into cellular allocation between two distinct processes. A log_2_ ratio > 1 indicates more than twice the number of growth‐related (*ftsZ*) reads compared to basal (RNase P) ones, whilst a log_2_ ratio < −1 suggests less than half. We applied mixed linear models to test for differences amongst all taxa, we found all eight aggregated taxonomic groups (Figure 2) to have different ratios across the experiment. For example, most clades exhibited negative fold differences (RNase *P* > *ftsZ*), which we interpret as indicating they put relatively less priority on growth, amongst which Flavobacterales was the lowest. On the other hand, *Rhodobacterales* showed the highest ratios (*ftsZ* > RNase P), which would suggest more energy allocation for growth and point to potential niche differentiation amongst these microorganisms. Additionally, we also tested for different fold change at day versus night time, and we found a significant day–night change for *Pelagibacterales*, *Synechococcales*, *Rhodobacterales* and *SAR86* (Figure 2).

## 4 Discussion

Microbial growth rate is a central parameter in developing mechanistic models of ecosystem function, yet measured rates offer a wide range of variation which ranges from minutes to months and even years (Hoehler and Jørgensen 2013; Weinstock et al. 2016). Due to its fundamental importance, genomic‐based methods have been developed to estimate these rates, such as codon usage bias (CUB) and peak‐to‐trough ratio (PTR) (Brown et al. 2016; Vieira‐Silva and Rocha 2010). CUB‐based methods aim to predict the maximum potential growth rates of microorganisms. They operate on the assumption that fast‐growing organisms tend to have genomes with more streamlined codon usage, which supports efficient protein synthesis (Weissman et al. 2021; Vieira‐Silva and Rocha 2010). In contrast, PTR ratios provide estimates of actual growth rates based on metagenomic data. This method relies on the idea that random metagenomic sequencing captures accurate coverage ratios between the origin and terminus of a genome, offering a direct measure of growth rate (Brown et al. 2016). Other methods based on quantifying metagenomic read fold change (Deulofeu‐Capo et al. 2024) (with cell count normalisation) or amplicon‐based changes (Fecskeová et al. 2021) (with standard spike‐ins) have been performed. These studies have found that prokaryotic doubling times are extremely diverse, ranging from approximately 0.3 to nearly 10 d^−1^, even amongst closely related taxa; more importantly, that variation increases at higher taxonomic definition, which was influenced by seasonal factors and top‐down controls (Deulofeu‐Capo et al. 2024; Fecskeová et al. 2021). Our approach, however, addresses a different question: how is growth distributed amongst the members of a microbial community? By focusing on this aspect, our work presents a way to bridge the gap between individual taxa contributions to classical bulk‐level assessments, much needed in light of the broad variation found by the previously mentioned methods.

Near coast and coastal environments are mesotrophic, dynamic and constantly face perturbations. Our experiment was deployed to assess microbial composition and processes before, during and after a spring bloom. The spring bloom off the Southern California coast has been shown to significantly affect the microbial community, causing rapid changes in microbial abundance and community composition (Needham and Fuhrman 2016; Needham et al. 2017, 2018). Our sampler was deployed before the bloom, but due to instrument failure 23 March–7 April, it missed sampling during the height of the bloom (5–6 April as indicated by satellite data, see Needham et al. (2018)) and was repaired in time (by 8 April) to capture the late bloom and its aftermath as the community returned to near its initial state (Needham et al. 2017).

Previous analyses of this experimental dataset focused on relative rRNA gene abundance, reflecting a combination of relative cell abundance and rRNA gene copy number per cell and relative rRNA abundance, which serves as a proxy for cumulative ribosome content and thus ‘potential activity’. However, this approach is susceptible to methodological biases (e.g., PCR amplification, incomplete taxonomic resolution) and introduces interpretive complexity in the derived inferences (Needham et al. 2018). To interpret our results, we consider that the profile of *ftsZ* expression consists of both abundance and identity information, where the cumulative expression per taxon represents the product of expression per cell times the number of cells, hence a large, slowly growing population could have the same amount of expression as a smaller but more actively expressing population. Even with this caveat in mind, the temporal shifts in *ftsZ* expression we observed during the bloom highlight the importance of considering short‐term dynamics in microbial community studies. Prior to and after the bloom, the microbial community was dominated by autotrophic and oligotrophic taxa such as *Synechococcales* and *Pelagibacterales*, which thrive in stable, low‐nutrient conditions. The ratios of *ftsZ*:RNase P that we calculated across the time series indicates that this dominance in cumulative growth might be derived from large population sizes rather that high activity per cell. The decline of the bloom triggered a significant shift towards heterotrophic bacteria, including *Rhodobacterales*, *SAR92* and *SAR86*. These taxa are likely capitalising on the organic matter released by decaying phytoplankton, reflecting a rapid response to the availability of fresh organic substrates. This finding is consistent with the well documented role of these heterotrophic bacteria in degrading phytoplankton derived organic matter following blooms (Teeling et al. 2016, 2012). Our study extends this understanding by providing a finer temporal resolution, capturing the day‐to‐day variations in microbial activity during the bloom's decline. Additionally, these results broadly match the amplicon‐based results from the same samples (Needham et al. 2018), however our approach provides a distinct layer of biological interpretation. Whilst rRNA:rRNA‐gene ratios primarily reflect cumulative ribosome content and potential activity, *ftsZ* expression specifically captures transcriptional allocation towards cellular division processes. Making it a more direct readout of microbial activity.

Interestingly, our analysis also revealed a significant proportion of *ftsZ* transcripts originating from chloroplasts within picoplanktonic eukaryotes. Whilst chloroplasts are typically not the focus of bacterial growth studies, their incidental inclusion in our metatranscriptomic analysis suggests that *ftsZ* may serve as a useful proxy for growth in both prokaryotic and eukaryotic phytoplankton. This finding aligns well with previous suggestions on factoring the many roles that eukaryotes play in marine productivity (Worden et al. 2015), however, we caution when interpreting *ftsZ* expression in eukaryotic organisms, as chloroplasts may not follow the same regulatory patterns as prokaryotic cells and are often recovered from a different size fraction (when samples are fractionated, as is commonly done).

Finally, the integration of *ftsZ* expression with RNase P normalisation adds a new dimension to our work. Whilst, admittedly, our approach (total RNA sequencing) limits sequencing depth because a large fraction of reads map to ribosomal RNA, this same approach allows us to obtain a comparatively unbiased picture of the community‐wide transcriptional profile. By comparing the relative expression of these two genes, we can infer how different taxa prioritise replication relative to other cellular functions. Our statistical analyses suggest that these differences are both taxa‐dependent and time‐point‐dependent, and are broadly consistent with previous environmental surveys (Deulofeu‐Capo et al. 2024), which are more comparable to our in situ observations than culture‐based studies. For example, our data suggest that *Flavobacterales* prioritise baseline transcriptional activity over rapid growth, whilst *Rhodobacterales* exhibit the opposite pattern. These observations are consistent with ASV‐based estimates of growth rates (Deulofeu‐Capo et al. 2024), where *Flavobacterales* displayed comparatively slower and more variable growth rates, whilst *Rhodobacterales* exhibited faster growth rates and appeared to be more strongly regulated by top‐down controls. This differentiation amongst microbial clades provides insights into their ecological roles and adaptations to changing environmental conditions. Similarly, the same previous work identified *Synechococcales* (Deulofeu‐Capo et al. 2024) as exhibiting strong seasonal and diel dependencies, patterns that were also captured in our analyses and described elsewhere (Binder and Chisholm 1995). Importantly, our results reflect in situ activities rather than laboratory‐based conditions (Long et al. 2021) or maximum predicted rates (Weissman et al. 2021); yet, they parallel these, as those microbes with higher *ftsZ:*RNase P ratios were identified as copiotrophs in a previous study (Weissman et al. 2021). Whilst still preliminary, our work suggests a potential framework for describing microbial community dynamics through first‐principles cellular biology.

## 5 Conclusions

In summary, our study highlights the importance of in situ transcriptomics in exploring fundamental questions of community dynamics. Decoupling presence from activity is essential for increasing our understanding of ecosystem function, particularly in dynamic coastal systems. We demonstrated the feasibility of our approach by analysing high‐resolution data and documenting how microbial activity shifts during phytoplankton blooms. Rather than directly estimating activity, our approach aims to describe how growth is partitioned amongst the members of a community. By doing so, it offers a bridge between fine phylogenetic individual contributions and bulk‐level measurements. Thus, future applications of our approach will offer insights that can lead to the development of testable hypotheses and more accurate models of planktonic food webs in changing oceans.

## Author Contributions

**Andrew Long:** resources, investigation. **Yuxuan Zou:** formal analysis, methodology, data curation. **J. Cesar Ignacio‐Espinoza:** conceptualization, methodology, formal analysis, writing – original draft, writing – review and editing, investigation. **Shengwei Hou:** resources, writing – review and editing, investigation. **David M. Needham:** resources, writing – review and editing. **Jed A. Fuhrman:** conceptualization, funding acquisition, writing – original draft, writing – review and editing.

## Funding

This work was supported by the Gordon and Betty Moore Foundation (3779), the Simons Foundation (549943), the National Natural Science Foundation of China (42276163, 42476109), and the Shenzhen Science and Technology Innovation Programme (JCYJ20220530115401003).

## Conflicts of Interest

The authors declare no conflicts of interest.

## Supporting information


**Figure S1:** Temporal dynamics of eukaryote‐assigned metatranscriptomic reads for *ftsZ* expression in the 0.22–1.0 μm size fraction. This figure shows the proportion of total *ftsZ* transcripts attributed to various picoeukaryotes, including *Bathycoccus* and *Micromonas*, as well as other eukaryotic taxa, across a time series from March 18 to May 1. The data were derived from chloroplasts of picoeukaryotes, which were captured in the 0.22–1.0 μm size fraction. Chloroplasts, having their own *ftsZ* gene, contribute to these reads. Larger eukaryotes are not represented due to size exclusion during sample processing. Because chloroplast ftsZ expression may not be directly comparable to prokaryotic *ftsZ* expression, these data are presented separately from the main figure (Figure 1).

**Table S1:** Detailed abundance of *ftsZ* sequencing fragments across identified microbial groups. This table presents the abundance of *ftsZ* sequencing fragments across all phylogenetic groups identified during the study. The table includes data at multiple taxonomic levels, from class down to families and genera, depending on the granularity of identification. Whilst Figure 1 highlights the most abundant orders, families and genera, this table provides the full breakdown of microbial contributions to *ftsZ* transcript abundance.

## Data Availability

The data that support the findings of this study are openly available in NCBI Bioproject at https://www.ncbi.nlm.nih.gov/bioproject, reference number PRJNA1166899.
