# Supplementary material for: In Situ Bacterioplankton Growth Partitioning by High‐Resolution Metatranscriptomics
Source: Environ Microbiol Rep. 2026 Jul 9;18(4):e70385. doi: 10.1111/1758-2229.70385 (PMC13349992; doi:10.1111/1758-2229.70385)
Supplement: Supplementary file 1 — Figure S1: Temporal dynamics of eukaryote‐assigned metatranscriptomic reads for ftsZ expression in the 0.22–1.0 μm size fraction. This figure shows the proportion of total ftsZ transcripts attributed to various picoeukaryotes, including Bathycoccus and Micromonas, as well as other eukaryotic taxa, across a time series from March 18 to May 1. The data were derived from chloroplasts of picoeukaryotes, which were captured in the 0.22–1.0 μm size fraction. Chloroplasts, having their own ftsZ gene, contribute to these reads. Larger eukaryotes are not represented due to size exclusion during sample processing. Because chloroplast ftsZ expression may not be directly comparable to prokaryotic ftsZ expression, these data are presented separately from the main figure (Figure 1). [file EMI4-18-e70385-s001.docx]

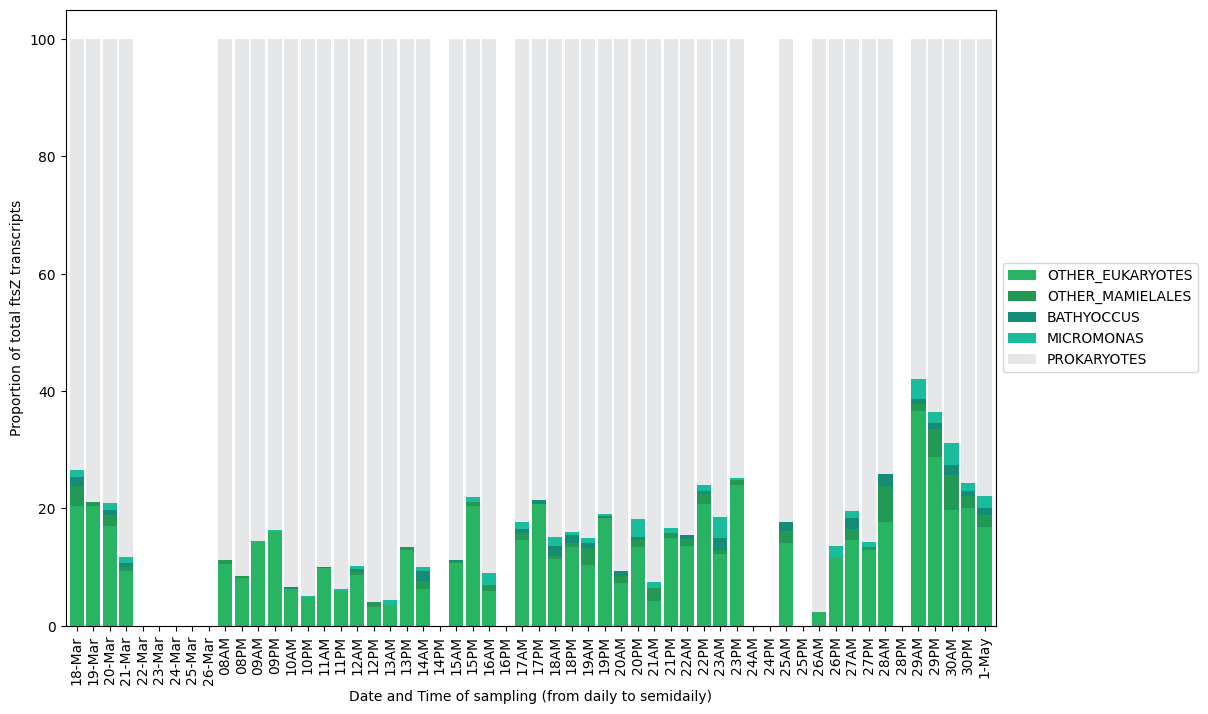


**Suppl. Figure 1.** **Temporal dynamics of eukaryote-assigned metatranscriptomic reads for *ftsZ* expression in the 0.22-1.0 µm size fraction.** This figure shows the proportion of total *ftsZ* transcripts attributed to various picoeukaryotes, including *Bathycoccus* and *Micromonas*, as well as other eukaryotic taxa, across a time series from March 18 to May 1. The data were derived from chloroplasts of picoeukaryotes, which were captured in the 0.22-1.0 µm size fraction. Chloroplasts, having their own *ftsZ* gene, contribute to these reads. Larger eukaryotes are not represented due to size exclusion during sample processing. Because chloroplast ftsZ expression may not be directly comparable to prokaryotic *ftsZ* expression, these data are presented separately from the main figure (Fig 1).
